# Effects of neurofeedback on standing postural control task with combined imagined and executed movements

**DOI:** 10.3389/fnins.2023.1199398

**Published:** 2023-07-07

**Authors:** Shun Sawai, Shoya Fujikawa, Chihiro Ohsumi, Ryu Ushio, Kosuke Tamura, Ryosuke Yamamoto, Yoshihiro Kai, Shin Murata, Keisuke Shima, Hideki Nakano

**Affiliations:** ^1^Graduate School of Health Sciences, Kyoto Tachibana University, Kyoto, Japan; ^2^Department of Rehabilitation, Kyoto Kuno Hospital, Kyoto, Japan; ^3^Department of Physical Therapy, Faculty of Health Sciences, Kyoto Tachibana University, Kyoto, Japan; ^4^Department of Rehabilitation, Tesseikai Neurosurgical Hospital, Shijonawate, Japan; ^5^Graduate School of Environment and Information Sciences, Yokohama National University, Yokohama, Japan

**Keywords:** motor imagery, neurofeedback, combination, motor execution, standing postural control

## Abstract

**Introduction:**

Motor imagery (MI) is a method of imagining movement without actual movement, and its use in combination with motor execution (ME) enhances the effects of motor learning. Neurofeedback (NFB) is another method that promotes the effects of MI. This study aimed to investigate the effects of NFB on combined MI and ME (MIME) training in a standing postural control task.

**Methods:**

Sixteen participants were randomly divided into MIME and MIME + NFB groups and performed 10 trials of a postural control task on an unstable board, with nine trials of MI in between. Electroencephalogram was assessed during MI, and the MIME + NFB group received neurofeedback on the degree of MI via auditory stimulation. A postural control task using an unstable board was performed before and after the MIME task, during which postural instability was evaluated.

**Results:**

Postural instability was reduced after the MIME task in both groups. In addition, the root mean square, which indicates the sway of the unstable board, was significantly reduced in the MIME + NFB group compared to that in the MIME group.

**Conclusion:**

Our results indicate that MIME training is effective for motor learning of standing postural control. Furthermore, when MI and ME are combined, the feedback on the degree of MI enhances the learning effect.

## 1. Introduction

Motor imagery (MI) is a cognitive process in which a person imagines that they are exercising without moving or tensing their muscles (Jeannerod, 1994). MI is widely applied in rehabilitation (Ferrer-Peña et al., 2021; Sen, 2021) and sports training (Mizuguchi et al., 2012) to improve movement accuracy, muscle power, and motor system flexibility (Ladda et al., 2021). MI shares some brain activities with motor execution (ME), including the primary motor cortex (M1), supplementary motor area (SMA), premotor area, parietal lobe, and cerebellum (Lotze et al., 1999; Hanakawa et al., 2003; Chepurova et al., 2022). The effects of MI have also been reported in standing postural control and have been found to improve the ability to control standing posture in the elderly (Nicholson et al., 2019), patients with stroke (Li et al., 2017), and those with Parkinson’s disease (Abraham et al., 2021). It has also been shown that motor learning is further promoted when MI and ME (Feltz and Landers, 1983) or MI and action observation (Taube et al., 2015) are combined. In addition, the effects of motor learning are higher with ME compared to MI (Driskell et al., 1994). When only ME is performed, fatigue and other physical stresses gradually occur. On the other hand, the exercise learning effect is smaller when only MI is performed compared to ME. MIME training has the advantage of providing an MI phase without actual exercise to reduce physical load, while ME provides a high motor learning effect (Bovend’eerdt et al., 2012; Wriessnegger et al., 2018). MIME training has also been shown to be effective in programs incorporating MI into ME or adding MI after ME (Schuster et al., 2012).

Additionally, neurofeedback (NFB) has recently attracted attention as a method of providing feedback to participants on whether MI is successful or not (Bai et al., 2014). NFB is a technique that improves exercise performance by providing feedback to participants on their brain activity during a task (Dettmers et al., 2016). It has been reported that electroencephalogram (EEG) activity related to MI involves desynchronization of μ/β rhythms in sensorimotor regions (McFarland et al., 2000) and suppression of μ waves in midline areas (Giromini et al., 2010). Therefore, the NFB of MI often feeds back event-related desynchronization (ERD) in the μ-wave band of the sensorimotor area (Pineda et al., 2003; Enomae et al., 2017). The use of this NFB increases the effects of MI (Seitz et al., 2009; Hamedi et al., 2016), and the effects of MI are reported in standing posture control (Fujimoto et al., 2017). These studies examined the effects of NFB on MI alone, but the effects of NFB in MIME training have not yet been studied. The use of NFB in MIME training is considered to have a high motor learning effect; therefore, clarifying this mechanism may contribute to the establishment of a motor learning program that can increase the effect of training. We hypothesized that the combined use of MIME and NFB would improve MI ability and standing postural control ability compared to MIME alone. Thus, in this study, we aimed to investigate the effects of applying NFB in MIME standing postural control training.

## 2. Methods

### 2.1. Participants

Sixteen healthy young men (20.94 ± 0.93 years) were recruited for the study. MI ability and brain function have been shown to be influenced by sex and age (Sullivan et al., 2009; Subirats et al., 2018). Therefore, to unify the characteristics of participants, only healthy young men were recruited in this study. Participants with a history of musculoskeletal or cognitive dysfunction and those with significant hearing impairment were excluded. The sample size for this study was calculated using G * Power (Kang, 2021). G power criteria were set as follows: test family, F tests; statistical test, analysis of variance (ANOVA); repeated measures, within-between interaction; effect size, 0.40; α error prob, 0.05; and power (1-β error prob), 0.80. The total sample size was calculated as 16. This study was conducted in accordance with the principles of the Declaration of Helsinki, and informed consent was obtained from all participants. This study was approved by the institutional ethics committee of Kyoto Tachibana University.

### 2.2. Study protocol

**Figure 1:**
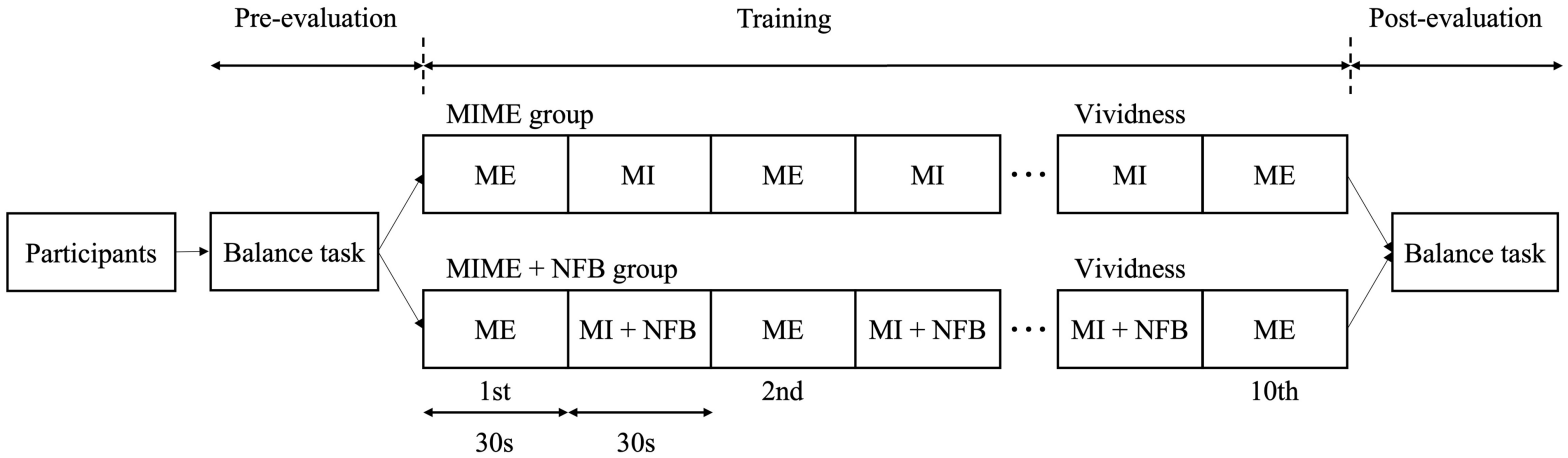
Study protocol. The participants were first evaluated in the standing postural control task twice in the pre-evaluation phase. Next, the participants were randomly assigned to the MIME (*n* = 8) and MIME+NFB (*n* = 8) groups. Ten ME trials of the standing postural control task were performed in the training phase, and nine MI trials were performed between each ME trial. Finally, in the post-evaluation phase, the standing postural control task was evaluated twice. MI, motor imagery; ME, motor execution; NFB, neurofeedback.

Participants were randomly classified into two groups: the MIME (*n* = 8) and MIME+NFB (*n* = 8) groups. The study was single-blinded, and the examiner was aware of the groups into which the participants were allocated. During the training phase, both groups performed MIME training with the standing postural control task, which consisted of 10 ME trials (30 s each) and nine MI trials (30 s each), with a standing postural control task between each ME trial. Five-second breaks were provided between each trial (Sawai et al., 2023). In the MIME+NFB group, EEG activity during MI in each trial was fed back by auditory stimulation. The same standing postural control task was performed twice, before and after training, to investigate the effect of the training. The vividness of MI was measured after the ninth MI trial (Figure 1).

### 2.3. Standing postural control task

**Figure 2:**
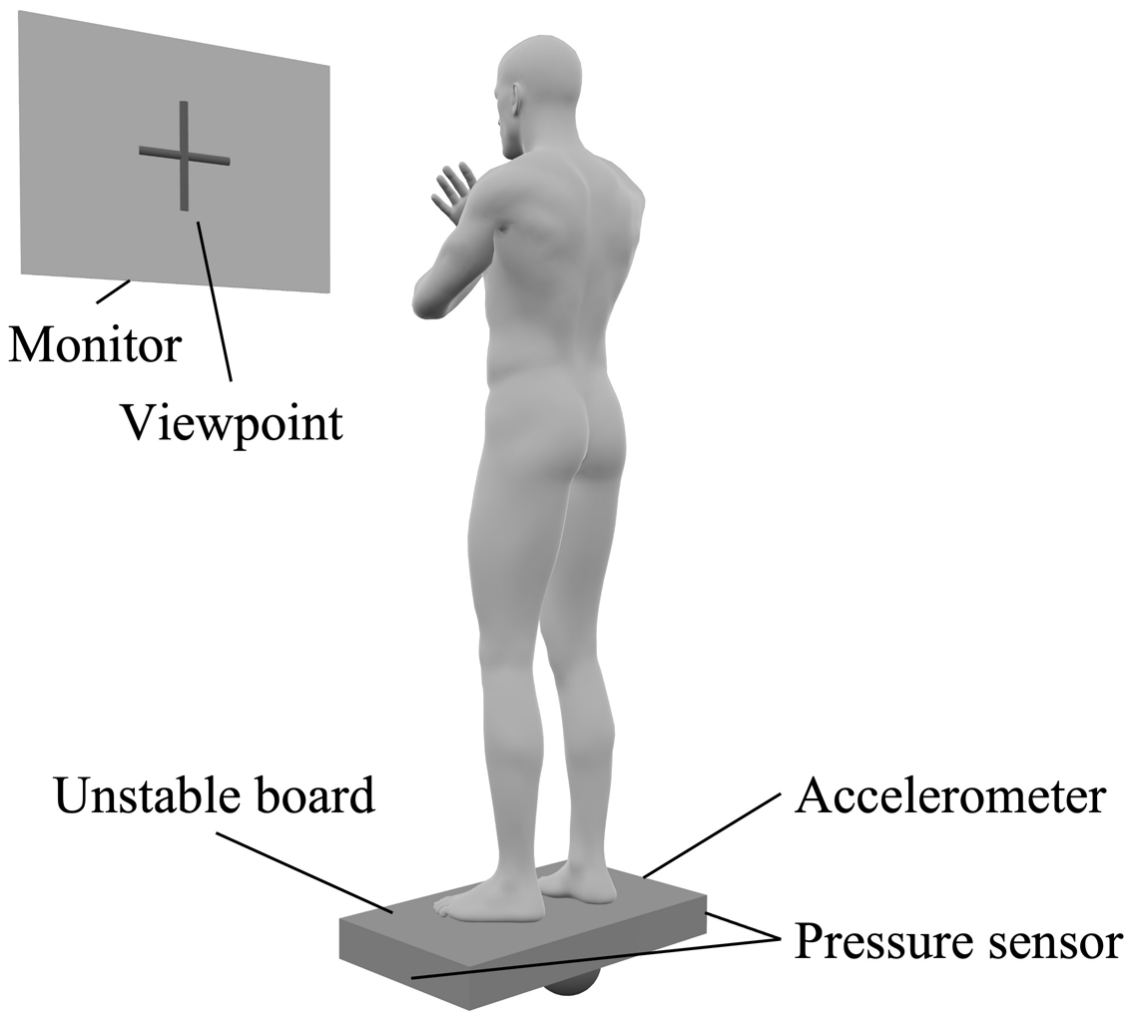
Standing postural control task setting. Participants stood on an unstable board with their arms crossed over their chest and the board held as horizontally as possible. Accelerometers and pressure sensors were placed on the unstable board to evaluate performance in the standing postural control task. A monitor was placed in front of the participant and a viewpoint was displayed on the monitor. We instructed participants to gaze at the viewpoint during the task.

In this study, participants stood on an unstable board (GB-100; OG Wellness Technologies Co., Ltd., Okayama, Japan) and performed a standing postural control task in which the unstable board was held as horizontally as possible. The unstable board was set to tilt only to the left and right, and not back and forth. Participants stood on the unstable board with their arms crossed over their chest. A monitor was placed 1.5 min front of each participant and a viewpoint was displayed at their eye level (Figure 2). At the start of the task, the unstable board was tilted 15° to the left, with the left edge of the board touching the floor (Supplementary Figure 1C). The participant then had to stabilize their posture to maintain the board as horizontal as possible when the task started. During the ME part of the MIME training, the participants performed 10 trials (30 s each) of the standing postural control task. Conversely, in the MI part of the MIME training, participants imagined the above-mentioned standing postural control task. The participants sat in a chair with a backrest and performed the kinesthetic imagery (first-person perspective) of the standing postural control task with closed eyes. Nine MI trials were performed between ME trials, with each trial lasting 30 s.

### 2.4. Neurofeedback

In the MIME+NFB group, EEG activity during MI in the MIME training was fed back via auditory stimulation. First, participants sat in a chair with a backrest, and resting EEG was measured. We instructed the participants to “relax and not think about anything” and encouraged them to rest. Then, we instructed the participants to “imagine the exercise as if you were performing the standing postural control task yourself” and encouraged them to imagine the exercise. EEG was measured using an electroencephalograph (EEG-1200, Nihon Kohden Corp., Tokyo, Japan) and active dry electrodes (Miyuki Giken, Co., Ltd., Tokyo, Japan). EEG was measured on 19 channels (Fp1, Fp2, F7, F3, Fz, F4, F8, T3, C3, Cz, C4, T4, T5, P3, Pz, P4, T6, O1, and O2) according to the international 10–20 method. Sampling was set at 1,000 Hz. The measured EEG data were analyzed using Microsoft Visual Studio (Microsoft Corp., Redmond, WA, United States) to evaluate resting EEG in the μ-wave band (8–13 Hz) in the sensorimotor regions (Cz). In the MI phase, EEG during imaging was measured and analyzed in the same way as resting EEG. The ERD was calculated from the resting EEG and the imagery EEG, and was fed back to the participants via auditory stimulation. The effects of NFB in the μ-wave band in the sensorimotor regions have been reported in previous studies (Pineda et al., 2003; Enomae et al., 2017).

The ERD value E(t) was calculated by measuring the resting-state EEG (R_rest) and the EEG at a certain time point t during MI (R_image (t), using the following formula.


$$E\left(t\right)=\frac{R_{rest}-R_{image}\left(t\right)}{R_{rest}}$$

ERD indicates a decrease in μ-wave activity during MI compared to that at the resting state. Therefore, E(t) takes (−∞ 1] and ERD occurs at (0 1].

This information was then fed back to participants via auditory stimulation. Because participants’ eyes were closed during NFB, auditory stimulation that does not require visual information was employed as feedback stimulation in this study. High-frequency sounds were provided when MI was good and sufficient ERD was occurring. Conversely, if the MI was poor and ERD was not sufficient, a low-frequency sound was provided. The frequency of the auditory feedback stimulus (S(t)) is calculated using the ERD (E(t)) at a given time (t), upper (E_max) and lower (E_min) limits of the ERD, and upper (S_min) and lower (S_max) limits of the frequency of the sound stimulus using the following formula.


$$S\left(t\right)=\left\{\begin{array}{l} 0\;\;\;\;\;\;\;\;\;\;\;\;\;\;\;\;\;\;\;\;\;\;\;\;\;\;\;\;\;\;\;\;\;\;\;\;\;\;\;\;\;\;\;\;\;\;\;\;\;\;\;\;\;\;\;\;\;\;\;\;\;\;\;\;\;\left(E\left(t\right)\leq E_{\min}\right)\\ \begin{array}{cc} S_{\min}+\left(S_{\max}-S_{\min}\right)\frac{E\left(t\right)}{E_{\min}} & \left(E_{\min}<E\left(t\right)\leq E_{\max}\right) \end{array}\\ \begin{array}{cc} S_{\max} & \;\;\;\;\;\;\;\;\;\;\;\;\;\;\;\;\;\;\;\;\;\;\;\;\;\;\;\;\;\;\;\;\;\;\;\;\;\;\;\;\;\;\;\;\;\;\;\;\;\left(E_{\max}<E\left(t\right)\right) \end{array} \end{array}\right.$$

Then, auditory feedback was represented by a continuous change in the auditory stimulation frequency. At this time, high-frequency sound was fed back during good MI and low-frequency sound was fed back during poor MI.

### 2.5. Measures

Accelerometers (AP-U166; Miyuki Giken, Co., Ltd., Tokyo, Japan) and pressure sensors (Miyuki Giken, Co., Ltd., Tokyo, Japan) were installed on the unstable board (Supplementary Figure 1), and a biometric signal recording device (MP-6100; Miyuki Giken, Co. Giken, Co., Ltd., Tokyo, Japan) was used to record the data. The accelerometer was placed at the right end of the unstable board, and the pressure sensors were placed at both ends of the back side of the unstable board (Supplementary Figure 1). The accelerometer had a detection range of ±200 m/s^2^, detection sensitivity of 10% ± 10 mV/G, frequency response of 0.8–800 Hz, and sampling rate of 1,000 Hz, enabling the detection of even minute changes in acceleration. In addition, the accelerometer was able to measure acceleration in three dimensions. Three outcomes that reflect the performance of the standing postural control task were calculated. The accelerometer calculated root mean square (RMS) from the measured acceleration data. From the acceleration data at each time (X) and the number of acceleration data (n), RMS was calculated using the following formula.


$$\mathrm{RMS}=\sqrt{\frac{X_{1}^{2}+X_{2}^{2}+\cdots X_{n-1}^{2}+X_{n}^{2}}{n}}$$

RMS is the average intensity of the waveform of the acceleration data and reflects the sway of the unstable board. The number of times the unstable board was in contact with the floor and the maximum horizontal holding time were calculated using a pressure sensor that detects the contact of the unstable board with the floor. The maximum horizontal holding time was defined as the maximum time the edge of the unstable board was maintained without contacting the floor during each 30-s standing postural control task. During the evaluation phase pre-and post-training, the standing postural control task was performed twice each time, and the average values were used for analysis. In addition, the difference between pre-training and post-training for each data set was also used in the analysis. Small values for the number of contacts and RMS indicate good performance. On the other hand, large values of the maximum horizontal holding time indicate good performance. Therefore, we calculated the number of contacts and RMS by subtracting post values from pre values and the maximum horizontal holding time by subtracting pre values from post values for the amount of change.

The subjective vividness of MI was evaluated using the visual analog scale (VAS) (Moriuchi et al., 2020). Participants self-evaluated the vividness of the motor images by marking a 100-mm horizontal line, where “0 = not at all” and “100 = very vivid image.” VAS was evaluated once at the end of the training phase.

### 2.6. Statistical analysis

First, the normality of each data set was confirmed using Shapiro–Wilk test. Then, independent *t*-test was used to compare the participant age and the postural control parameters at baseline between groups. Two-way mixed model ANOVA using two factors, namely the group (MIME and MIME+NFB groups) and time (pre and post), was used to examine the significant main effects and interactions. Subsequently, Bonferroni *post hoc* test was used for multiple comparisons. In addition, Mann–Whitney *U* test was used to compare the amount of change in each measure between pre-and post-training, and the VAS in training. We used SPSS version 28.0 (IBM Corp., Armonk, NY, United States) for statistical analyses. The significance level was set at *p* < 0.05.

## 3. Results

**Figure 3:**
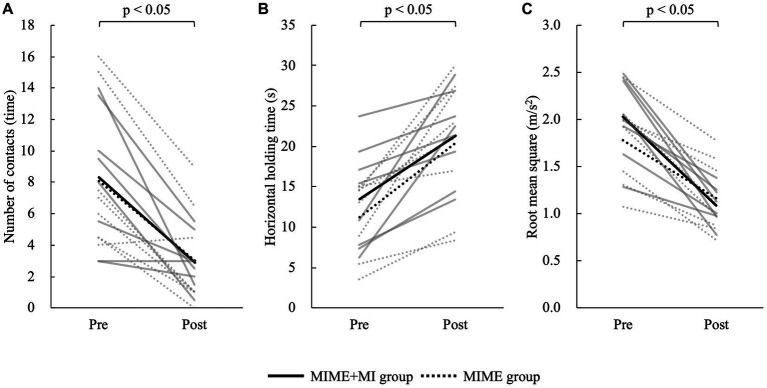
Comparison of each data pre-and post-training. The solid line shows the MIME+NFB group and the dotted line shows the MIME group. The gray line shows the data for each participant and the black line shows the mean value for each group. **(A)** Comparison of the number of contacts with the floor pre-and post-training; the number of contacts decreased significantly in both MIME and MIME+NFB groups after training (*p* < 0.05). **(B)** Comparison of maximum horizontal holding time pre-and post-training; both MIME and MIME+NFB groups significantly prolonged their maximum horizontal holding time after training (*p* < 0.05). **(C)** Comparison of RMS pre-and post-training; both MIME and MIME+NFB groups had significantly decreased RMS after training (*p* < 0.05). MI, motor imagery; ME, motor execution; NFB, neurofeedback; RMS, root mean square.

**Figure 4:**
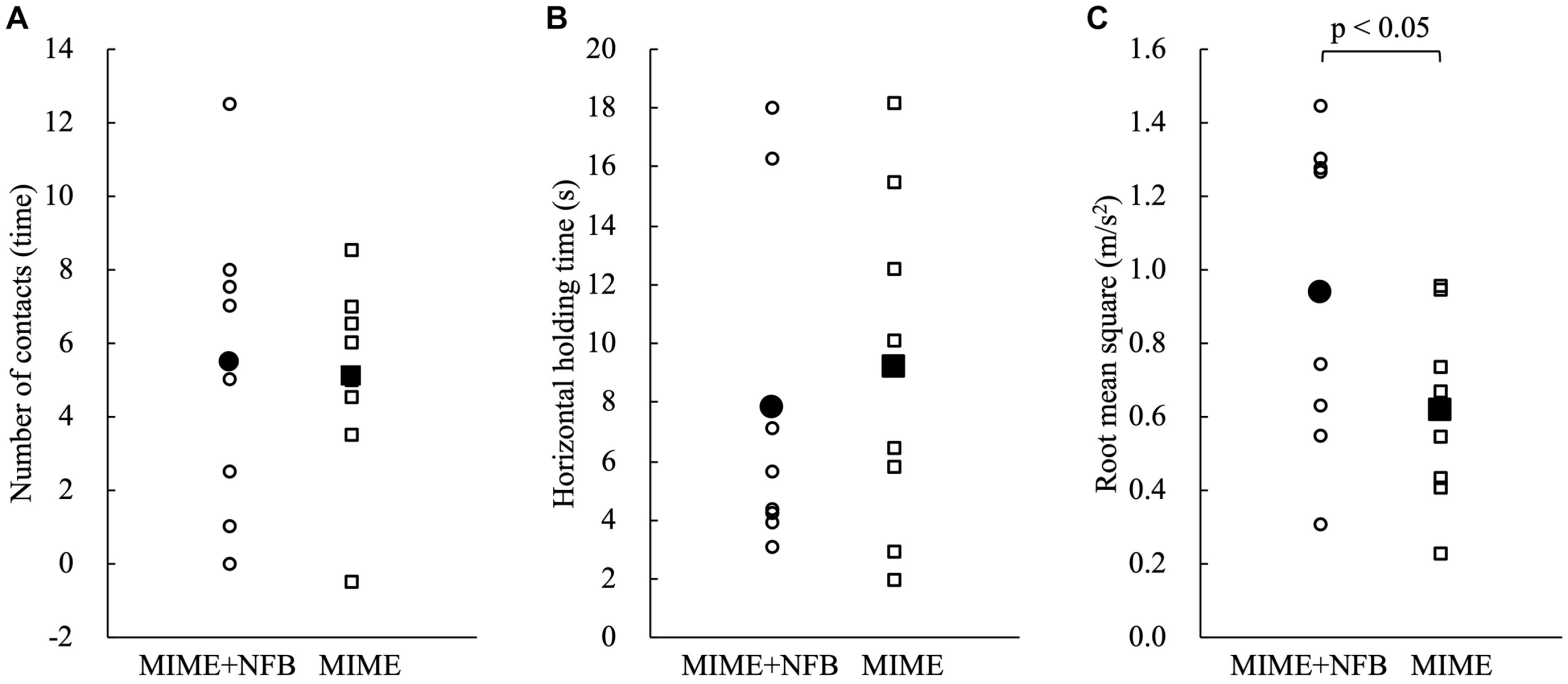
Comparison of the amount of change pre-and post-training. Circles indicate the MIME+NFB group, and squares indicate the MIME group. The white plots show the data for each participant, and the black plots show the mean values for each group. Small values for the number of contacts and RMS indicate good performance. On the other hand, large values of maximum horizontal holding time indicate good performance. We calculated the number of contacts and RMS by subtracting post from pre and the maximum horizontal holding time by subtracting pre from post for the amount of change. Therefore, the large change in all parameters indicates a significant improvement in performance. **(A)** There was no significant difference between the MIME and MIME+NFB groups in the number of contacts with the floor pre-and post-training (*p* > 0.05). **(B)** There was no significant difference between the MIME and MIME+NFB groups in the maximum horizontal holding time pre-and post-training (*p* > 0.05). **(C)** The pre-and post-training changes in RMS were significantly higher in the MIME+NFB group than those in the MIME group (*p* < 0.05). MI, motor imagery; ME, motor execution; NFB, neurofeedback; RMS, root mean square.

First, the Shapiro–Wilk test showed that all data except VAS were normally distributed (*p* > 0.05). Then, independent *t*-test showed no significant group differences in participant age and the postural control parameters at baseline (*p* > 0.05). The two-way mixed model ANOVA showed that the number of contacts with the floor (*F* = 35.60, *p* < 0.05), maximum horizontal holding time (*F* = 33.16, *p* < 0.05), and RMS (*F* = 76.41, *p* < 0.05) had significant main effects on the time factor (Figure 3). However, these postural control parameters showed no significant main effects on the group factor and interaction between group and time (*p* > 0.05). The Bonferroni *post hoc* test results also revealed that the number of contacts with the floor was significantly lower after training (*p* < 0.05), maximum horizontal holding time was significantly longer after training (*p* < 0.05), and RMS was significantly smaller after training (*p* < 0.05). The number of contacts with the floor improved by 65.18% ± 36.15% in the MIME group and 55.60% ± 30.83% in the MIME+NFB group. Maximum holding time improved by 92.65% ± 62.91% in the MIME group and 84.76% ± 87.31% in the MIME+NFB group. Furthermore, RMS improved by 34.47% ± 12.49% in the MIME group and 44.23% ± 14.36% in the MIME+NFB group. Additionally, the Mann–Whitney *U* test results showed that the change in RMS was significantly higher in the MIME+NFB group than that in the MIME group (*p* < 0.05). Conversely, there was no significant difference in the number of contacts with the floor and maximum horizontal holding time between these groups (*p* > 0.05; Figure 4). VAS was 48.88 ± 12.25 for the MIME group and 73.00 ± 9.68 for the MIME+NFB group, with the VAS for the MIME+NFB group being significantly higher than that for the MIME group (*p* < 0.05) (Supplementary Figure 2).

## 4. Discussion

This study examined the effects of applying NFB during MIME training for standing postural control. The results showed that both the MIME and MIME+NFB groups had a decrease in RMS and the number of contacts with the floor. Additionally, the maximum horizontal holding time after training was prolonged in both groups. Moreover, a comparison of the amount of change pre-and post-training showed that the MIME+NFB group had a significantly higher change in RMS than the MIME group. Furthermore, the vividness of MI was significantly higher in the MIME+NFB group than that in the MIME group. These results suggest that MIME training improves the ability to control standing posture and that the application of NFB during MIME training promotes the motor learning effect.

Both MIME and MIME+NFB groups showed a decrease in the number of contacts with the floor and RMS and an increase in maximum horizontal holding time after training. Therefore, these results can be explained by previous studies examining the motor learning effects of MI, ME, and MIME. Motor learning by ME has been reported in many cases, and it has been shown that performance is improved by exercise repetition (Halsband and Freund, 1993; Krakauer et al., 2019). Similar results have been reported for standing postural control tasks (Giboin et al., 2019; Keller et al., 2023). In addition, motor learning with MI was also shown to have positive effects in previous studies (Sen, 2021). Furthermore, MIME training using a combination of these two methods has been found to have a high motor learning effect (Bovend’eerdt et al., 2012; Wriessnegger et al., 2018). In this study, both MIME and MIME+NFB groups showed improvements in performance in the standing postural control task after MIME training. These results indicate that MIME training is effective for motor learning of standing postural control.

The change in RMS pre-and post-training was significantly higher in the MIME+NFB group compared to that in the MIME group. RMS reflects the level of instability of the unstable board, with smaller values indicating better performance. In addition, a higher change in RMS indicates a smaller RMS after training, which also indicates better performance. Therefore, the results of this study suggest that applying NFB in MIME training can better promote the effects of motor learning. NFB in MI has been reported to improve the effects of MI (Seitz et al., 2009; Hamedi et al., 2016). However, the effect of NFB in this study may have occurred because MI and ME were used together. It has been reported that MIME training improves the accuracy of MI, as MI is performed based on information obtained from ME (Wriessnegger et al., 2014). This suggests that ME may promote MI. In this study, NFB with MI promoted by ME may have improved performance as a result of the increased effect obtained from MI. Since there are no previous studies with the same tasks and conditions as those in this study, it is difficult to directly compare the amount of change to that in other studies. However, it is clear that MI, ME, and MIME improve performance and facilitate motor learning (Feltz and Landers, 1983; Schuster et al., 2012; Ladda et al., 2021), and this study suggests that adding NFB to MIME may further improve these effects. Balance tasks such as those used in this study are used in sports training and rehabilitation. Therefore, the results of this study may be applied to balance training in sports training and rehabilitation to improve its effectiveness.

In previous studies examining the effects of NFB on MI, the brain activity states of the M1 (Pineda et al., 2003; Enomae et al., 2017; Makary et al., 2017; Yang et al., 2021), SMA (Pineda et al., 2003; Enomae et al., 2017; Al-Wasity et al., 2021), prefrontal area (Ota et al., 2020), and premotor area (Xie et al., 2015) were fed back to participants. This was because changes in performance due to MI result from activation of motor-related areas, cerebellum, parietal lobe, and visual cortex (Zhang et al., 2012; Xu et al., 2014). In this study, feedback was provided based on the activity of sensorimotor regions, including the M1 and SMA. Among these, the M1 is related to attention, motor learning, motor integration, motor inhibition, and motor response (Bhattacharjee et al., 2021). Moreover, the SMA is important for standing postural control in the medial and lateral directions (Herold et al., 2017), and it has been shown that feedback based on the brain activity state of the SMA can improve standing postural control (Fujimoto et al., 2017). The results of this study suggest that ERD feedback from the sensorimotor regions involved in standing postural control and motor learning may improve performance as a result of NFB-induced MI facilitation.

VAS was significantly higher in the MIME+NFB group than that in the MIME group. It is suggested that NFB for MI corresponds to knowledge of result in motor learning (Mihara et al., 2012), and that the participants’ recognition of brain activity during MI may have improved the subjective vividness of MI. Although ME has also been shown to provide knowledge of the results about MI (Guillot et al., 2012), it is not possible to perform MI and ME simultaneously. Simultaneous implementation of MI and NFB may have improved subjective vividness of MI by feeding back real-time knowledge of results.

There are several limitations to this study. First, this study did not examine the effects of NFB on MI alone. Future studies should compare MIME, MIME+NFB, and MI + NFB groups to determine the extent to which NFB and ME contribute to MI promotion. Second, this study failed to examine the effect of MIME+NFB on EEG, which has been shown to be an objective measure of MI ability (Pineda et al., 2003; Enomae et al., 2017). EEG may reveal detailed effects of MIME+NFB. Third, this study uses accelerometers to measure the acceleration of the unstable board, which is used to evaluate the standing posture control performance of maintaining the board horizontal. However, the accelerometer only detects changes in the board’s tilt and does not consider the degree to which the board is held in a tilted position. Future research may reveal detailed standing posture control performance by evaluating the three-dimensional tilt of the board with a gyro sensor. Fourth, this study did not examine the retention effect of motor learning. In future studies, it will also be necessary to investigate the extent to which the effect of NFB in MIME training is sustained. By investigating these effects, we hope to increase the applicability of NFB in MIME training to fields such as rehabilitation and sports training. Fifth, the participants in this study were limited to healthy young men. Therefore, there are barriers to generalizing the results of this study. Future studies could broadly apply these results by expanding the target population to include older adults and women. Another possibility is that the effect of NFB may have been smaller in this study because the participants were healthy young men with high standing postural control ability. It may be possible to clarify the effect of NFB in detail by targeting elderly people and patients with low standing postural control ability. In addition, by examining the effects on stroke patients and athletes, rehabilitation and sports training using MIME+NFB can be devised.

In conclusion, we investigated the effects of applying NFB in MIME standing postural control training. Our results suggest that MIME training is effective for motor learning of standing postural control and that the application of NFB to MIME training can promote motor learning. The effect of NFB in MIME training has the potential to be widely applied to rehabilitation, sports training, and other fields.

## Data availability statement

The raw data supporting the conclusions of this article will be made available by the authors, without undue reservation.

## Ethics statement

The studies involving human participants were reviewed and approved by the Institutional Ethics Committee of Kyoto Tachibana University. The patients/participants provided their written informed consent to participate in this study.

## Author contributions

SS and HN: conceptualization. SS, SF, CO, RU, KT, RY, YK, SM, and HN: methodology. SS, SF, CO, RU, KT, RY, and HN: validation. SS, SF, CO, and HN: formal analysis, data curation, writing—original draft preparation, and visualization. SS, SF, CO, RU, KT, RY, YK, SM, KS, and HN: investigation and writing—review and editing. SM, KS, and HN: resources. HN: supervision, project administration, and funding acquisition. All authors contributed to manuscript revision and read and approved the submitted version.

## Funding

This work was supported by JSPS KAKENHI Grant Numbers JP20K11173 and JP23K10417, and the Sasakawa Scientific Research Grant from The Japan Science Society.

## Conflict of interest

The authors declare that the research was conducted in the absence of any commercial or financial relationships that could be construed as a potential conflict of interest.

## Publisher’s note

All claims expressed in this article are solely those of the authors and do not necessarily represent those of their affiliated organizations, or those of the publisher, the editors and the reviewers. Any product that may be evaluated in this article, or claim that may be made by its manufacturer, is not guaranteed or endorsed by the publisher.
