# Supplementary material for: Effects of neurofeedback on standing postural control task with combined imagined and executed movements
Source: Front Neurosci. 2023 Jul 7;17:1199398. doi: 10.3389/fnins.2023.1199398 (PMC10360181; doi:10.3389/fnins.2023.1199398)
Supplement: Supplementary file 1 [file Data_Sheet_1.pdf]

Supplementary Material

Effects of Neurofeedback on Standing Postural Control Task with Combined Imagined and Executed Movements

Shun Sawai, Shoya Fujikawa, Chihiro Ohsumi, Ryu Ushio, Kosuke Tamura, Ryosuke Yamamoto, Yoshihiro Kai, Shin Murata, Keisuke Shima, Hideki Nakano*

*** Correspondence:** Hideki Nakano: nakano-h@tachibana-u.ac.jp

1 Supplementary Figures

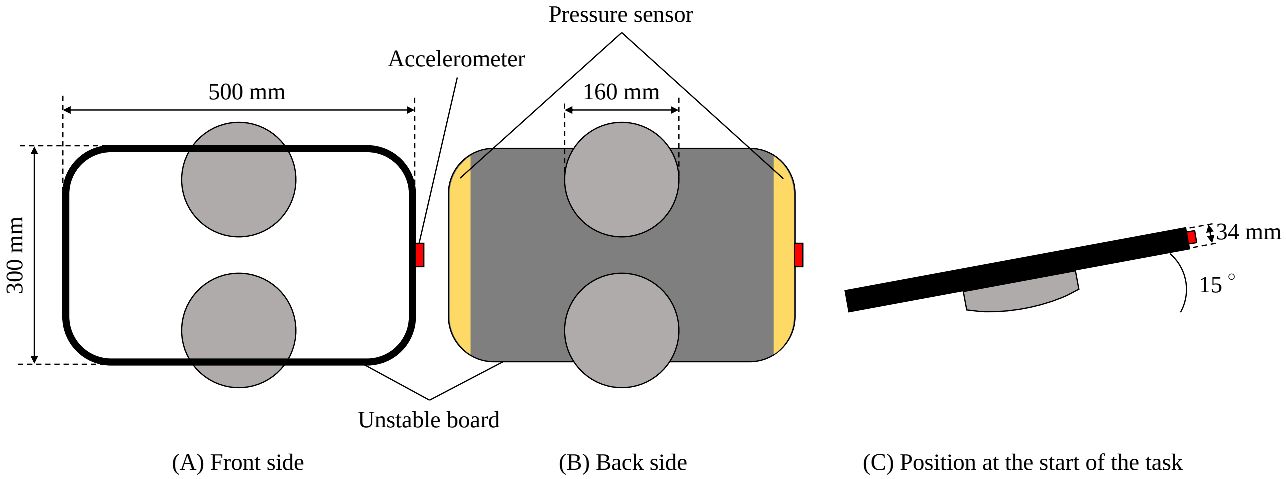

Supplementary Figure 1. Experimental setup of the unstable board

The unstable board was 300 mm long, 500 mm wide, and 34 mm high. The board was set to tilt only to the left or right. (A) The front face of the unstable board is shown. An accelerometer was attached to the right edge of the board. (B) Back side of the unstable board. Pressure sensors were mounted at both ends of the board. (C) The position of the board at the start of the task. The board was tilted to the left and started the task from the position where the edge of the board touched the floor. At this time, the board was tilted 15° to the left from the floor.

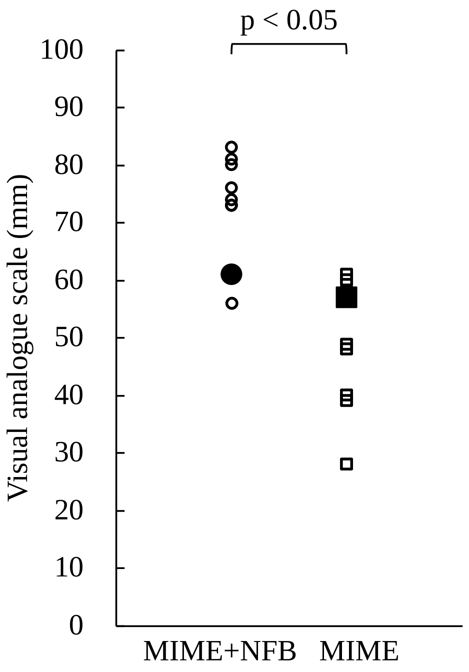

Supplementary Figure 2. Comparison of the VAS between groups

Circles indicate the MIME+NFB group, and squares indicate the MIME group. The white plots show the data for each participant, and the black plots show the mean values for each group. VAS was significantly higher in the MIME+NFB group than that in the MIME group ($p < 0.05$).

MI, motor imagery; ME, motor execution; NFB, neurofeedback; VAS, visual analogue scale
